# Bronchopulmonary Dysplasia Early Changes Leading to Long-Term Consequences

**DOI:** 10.3389/fmed.2015.00002

**Published:** 2015-02-12

**Authors:** Anne Hilgendorff, Michael A. O’Reilly

**Affiliations:** ^1^Comprehensive Pneumology Center, Helmholtz Zentrum München, Member of the German Center for Lung Research (DZL), Munich, Germany; ^2^Neonatology, Perinatal Center Grosshadern, Dr. von Hauner Children’s Hospital, Ludwig-Maximilians University, Munich, Germany; ^3^Department of Pediatrics, School of Medicine and Dentistry, The University of Rochester, Rochester, NY, USA

**Keywords:** bronchopulmonary dysplasia, neonatal chronic lung disease, lung development, long-term consequences, hyperoxia, mechanical ventilation, inflammation, animal models

## Abstract

Neonatal chronic lung disease, i.e., bronchopulmonary dysplasia, is characterized by impaired pulmonary development resulting from the impact of different risk factors including infections, hyperoxia, and mechanical ventilation on the immature lung. Remodeling of the extracellular matrix, apoptosis as well as altered growth factor signaling characterize the disease. The immediate consequences of these early insults have been studied in different animal models supported by results from *in vitro* approaches leading to the successful application of some findings to the clinical setting in the past. Nonetheless, existing information about long-term consequences of the identified early and most likely sustained changes to the developing lung is limited. Interesting results point towards a tremendous impact of these early injuries on the pulmonary repair capacity as well as aging related processes in the adult lung.

## Clinical Background

The neonatal form of chronic lung disease (CLD), also known as bronchopulmonary dysplasia (BPD), is one of the most common forms of CLD in early infancy. The disease results from the impact of different risk factors on the undeveloped neonatal lung and is associated with a significantly increased risk for pulmonary and neurologic impairment persisting into adulthood in the cohort of formerly preterm infants (1). Defined by the need for supplemental oxygen and/or ventilatory support for >28 days, or beyond 36 weeks post-menstrual age (PMA), the disease can be classified into three different severity grades (mild, moderate, severe) (1).

The incidence of BPD is reported to be up to 77% in infants born at <32 weeks of gestation with a birth weight below 1 kg (2–4) but varies between newborn care centers, reflecting differences in patient population and infant management practices (2, 5–7). Even significant improvements in perinatal care including surfactant treatment, administration of antenatal corticosteroids, and improvement of invasive and non-invasive ventilation strategies could not significantly alter the incidence of long-term sequelae associated with the disease in the most immature infants (8).

Clinically, this form of CLD presents with hypoxemia leading to the need for supplemental O_2_ as well as hypercapnia, reflecting impaired respiratory gas exchange and alveolar hypoventilation, resulting in a mismatch of ventilation and perfusion (9). Lung function is characterized by diminished compliance, tachypnea, increased minute ventilation, and work of breathing and can be accompanied by an increase in lung microvascular filtration pressure leading to interstitial pulmonary edema (10). As a result of increased respiratory tract resistance and hyper-reactive airways, episodic bronchoconstriction and cyanosis can be observed (11), with early lung function impairment indicating more severe disease at term (12). The increased lung vascular resistance, typically associated with impaired responsiveness to inhaled nitric oxide and other vasodilators, can progress to reversible or sustained pulmonary hypertension and right heart failure (13, 14).

As a trigger for the onset of these pathophysiologic processes, large clinical trials have identified important risk factors (15–20). Besides postnatal infections, the requirement for prolonged assisted ventilation to treat acute respiratory failure caused by primary surfactant deficiency and the need for oxygen supplementation is known to injure the structural and functional immature lung (21–24). Here, the use of large tidal volumes and high inflation pressures, in concert with the magnitude and duration of exposure to supplemental oxygen, are major risk factors for disease development (25, 26). The occurence of pulmonary complications, e.g., air leaks, interstitial emphysema, and pneumothoraces, further increase the risk (25). With respect to postnatal growth and development, poor nutritional support, vitamin deficiency as well as insufficient adrenal and thyroid hormone release in the very premature infant is known to significantly contribute to adverse pulmonary outcome (27–29).

These postnatal stressors are known to act beyond the background of both prenatal as well as genetic risk factors influencing the capacity of the developing lung to respond to the indicated postnatal injuries:

Intrauterine growth retardation increases the risk of BPD three to fourfold (30–34), most likely through impaired alveolar and vascular growth associated with altered growth factor signaling (35). The prenatal impact of cytokines, in the presence, i.e., chorioamnionitis or absence, i.e., fetal inflammatory response syndrome of other signs of infection is known to prime the lung for a pathologic response to postnatal injury, thereby contributing to BPD development (36).

Regarding the impact of the genetic background, a study investigating twin preterm infants found genetic factors to account for 53% of the variance in liability for BPD (37). Several potential candidate genes have been associated with BPD, where genetic variants predisposing to BPD are usually polymorphisms, which are not causative, but have been shown to increase disease susceptibility. Genetic abnormalities include variations affecting the surfactant system or the innate immune response (38, 39). Such conclusions are consistent with a genome-wide association study involving more than 1700 very low birth weight infants that was not able to link specific genomic loci or pathways with BPD (40). Instead, gene linkage studies found that one of the corticotropin-releasing hormone receptors (CRHR1) or cytochrome P450 2E1 (CYP2E1) in the preterm infant, and ectonucleotide pyrophosphatase 1 (ENPP1), insulin-like growth factor binding protein 3 (IFGBP3), 7-dehydrocholesterol reductase (DHCR7), or tumor necrosis factor-associated factor 2 (TRAF2) in the mother were highly associated with preterm birth (41). Nonetheless, gestational age at birth still remains one of the most accurate ways to predict the incidence of BPD (42). In addition, male preterm infants are at a higher risk for the development of long-term impairment, including BPD (43), and premature changes to hormonal regulation have been discussed as an underlying cause (44). The effect of gender seems to be different with respect to the adult population, as female adult BPD patients are more severely affected with respect to developing long-term pulmonary impairment (45).

Animal models were instrumental in elucidating some of the underlying mechanisms by which the indicated risk factors resulted in profound and durable structural changes in the developing lung and will be discussed in the pathophysiologic context.

## Pathophysiologic Characteristics

With respect to histopathology, neonatal CLD is characterized by impaired alveolarization and vascularization (1). Alveolarization in humans begins during late fetal development and continues into early childhood (46). Although continued growth of new alveoli was observed in rhesus monkeys up into adulthood (47), there is increasing evidence in both humans and rodents suggesting alveolar development to occur in two phases (46, 48, 49). The majority of alveoli develops in the early phase and occurs as double micro-capillaries mature into a single capillary network. This phase is followed by ongoing alveolar growth arising from existing alveoli. Knowledge about these two distinct phases in alveolar development is critical to design therapeutic approaches aiming to promote alveolar development in the injured neonatal lung. As a well-known example, postnatal corticosteroids have been used to promote lung function, facilitating extubation and thereby trying to prevent or ameliorate BPD development. Although their anti-inflammatory effects are potent (50), dexamethasone administered to newborn rats has been shown to cause alveolar thinning and structural simplification, presumably by inhibiting the early phase of alveolar development (51). Especially, the impact of postnatal steroid therapy on extracellular matrix (ECM) composition as observed in rats may account for long-term effects (52). Not only is the window of lung development a critical variable for the decision on postnatal steroid therapy, but the increased risk for gastrointestinal bleeding, cardiovascular disease, and cerebral palsy in infants that had received steroid therapy (53, 54), strongly limits dose, time-point, and length of treatment.

Besides the continuous changes in pre- and postnatal treatment strategies in preterm care, the altered histo- and pathophysiological picture of BPD over the last decades is mainly due to the degree of immaturity in the preterm cohort studied. As the disease was largely an atelectatic fibrosing disease attributed to persistent oxygen exposure in late preterm infants (>34–36 weeks) in the pre-surfactant era (55), the implementation of exogenous surfactant administration as well as improved ventilation strategies and the induction of lung maturation by prenatal steroids significantly increased the survival of infants as immature as 23–24 weeks of gestation. Although the amount of interstitial fibrosis is substantially less in these infants and tends to be more diffuse when compared to histopathology in the pre-surfactant era, extensive ECM remodeling together with increased smooth muscle in small pulmonary arteries and airways (56) remains a key finding in the diseased lung.

Many studies have shown that characteristic inflammatory changes and altered growth factor signaling precede and accompany these changes to the pulmonary scaffold that may not only hinder physiologic lung development but also transfer its long-term effects into adulthood. The characteristic degradation of lung elastin that accompanies its pathologic distribution pattern in infants who later acquire BPD manifests in increased urinary excretion of desmosin, a breakdown product of the mature elastic fiber, preceded and paralleled by an associated increase in protease activity (57–62). Nonetheless, studies in an inflammatory model of BPD have shown that a delicate balance of protease activity is critical for normal lung development, as complete matrix-metalloproteinase deficiency worsened lung injury (63). Experimental studies in rodents recapitulated the changes to the ECM and allowed further insight into disease relevant pathophysiology, linking ECM remodeling to apoptosis, inflammation, and growth factor signaling (64–67). First attempts were made in order to therapeutically prevent ECM degradation, thereby preserving lung growth in the presence of mechanical ventilation (68, 69).

Pulmonary inflammation induced by both non-infectious processes, such as positive-pressure ventilation or oxygen therapy aggravated by primary surfactant deficiency, or patent ductus arteriosus, as well as pre- or postnatal infections play an important role in the translation of different injury mechanisms to structural and functional changes as well as in the aggravation of ongoing pathologic processes in the developing lung (18, 22, 70, 71). The characteristic initial influx of neutrophils into the lung is accompanied and followed by increased numbers of macrophages in the course of the disease (32, 72, 73). Explaining the perpetuation of the inflammatory response, animal studies indicate that lung injury leading to ECM remodeling or early alveolar epithelial dysfunction further promotes lung inflammation (68, 74). In contrast, inflammation in fetal sheep infected with *Ureaplasma parvum*, a common microorganism present in chorioaminionitis, did not affect lung development (75), suggesting that inflammation acts in concert with other risk factors to provoke the development of BPD.

The release of cytokines and disturbance of growth factor signaling, [e.g., transforming growth factor (TGF)-β], leads to the activation of different transcription factors, and results in a characteristic increase in apoptosis affecting all different cell types (76). With relevance to the mesenchymal cells, the platelet derived growth factor-α, characterizing the myofibroblast driving secondary crest formation, and the fibroblast growth factor family [for review see Ref. (77)] are known to be of importance. The presence of dysmorphic capillaries is related to an altered pattern of angiogenic growth factors such as reduced expression of the vascular endothelial growth factor (VEGF) and its receptors (78–80) in the lung, accompanied by diminished endothelial nitric-oxide synthase (eNOS) and soluble guanylate cyclase (sGC) in lung blood vessels and airways (81, 82). These changes contribute to subsequent development of pulmonary hypertension and impaired lung lymphatic drainage (13, 14).

The growing evidence that prenatal factors impact on the incidence of childhood asthma (83) further underlines the importance of studies focusing on the role of prenatal variables in respiratory development following premature birth including the broad issue of maternal health. Suggested by a study in newborn mice exposed to hyperoxia after maternal exposure to LPS during pregnancy, the enhancement of cardiovascular disease in the animals points to an impact of chorioamnionitis not only on surfactant production but also on cardiovascular health in children born premature undergoing early oxygen treatment (84).

Relative deficiencies of anti-oxidants and inhibitors of proteolytic enzymes render the very immature lung, especially vulnerable to the effects of toxic oxygen metabolites and proteases released by the ECM, resident lung cells, or activated neutrophils and macrophages (85–88). Elevated urinary malondialdehyde concentrations in the first week of life, generated by peroxidation of lipid membranes after oxidant-mediated injury, were correlated with the risk for oxygen radical diseases including BPD (89). The association of genetic polymorphisms of the superoxide dismutase with the development of BPD underlines the importance of a balanced redox system (90).

**Figure 1:**
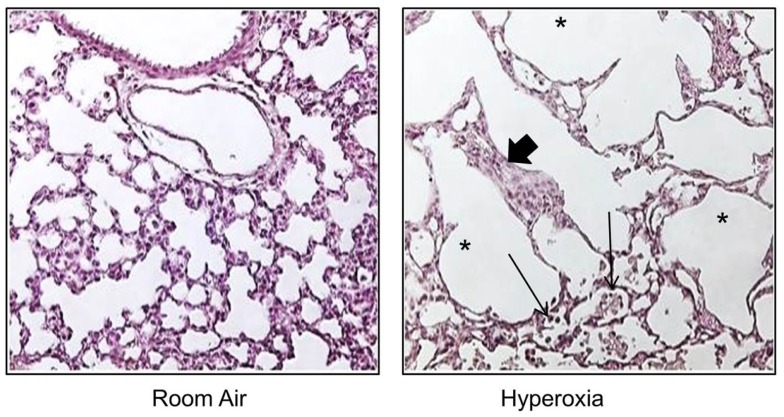
**Neonatal hyperoxia disrupts postnatal alveolar development in the lung**. Representative tissue slides (H&E stains) of newborn mouse lung exposed to room air or 100% oxygen from birth to PN10. Thickened alveolar septae (thick arrow), inflammatory cells (thin arrow), and simplified alveoli (asterisks).

To understand how oxygen affects lung development, non-human primates, preterm sheep, newborn guinea pigs, and newborn rodents have been exposed to excessive levels of oxygen [for review in Ref. (91)]. Early-life oxygen exposure leads to many characteristic pathologic features of the so called “new” BPD, including inhibition of microvascular development, alveolar simplification, inflammation, and mild interstitial thickening [Figure 1 and Ref. (92, 93)]. It also recapitulates many diseases in children who were born preterm, including altered host response to respiratory viral infections, mild cognitive changes, and cardiovascular disease. Despite the widespread use of hyperoxia as a tool to study BPD, a codified model of oxygen exposure has yet to be established, making the extrapolation of outcomes between different investigators using different doses and durations of hyperoxia on different developmental windows possible (91). Today, preterm infants are often exposed to excess oxygen during the saccular stage of lung development, and discharged breathing room air when entering the first phase of completion in alveolarization. In order to mimic these clinically relevant conditions, the influence of oxygen on saccular stages in lung development, i.e., mouse E17.5 to PN should be separated from its impact on the phase of alveolarization, i.e., mouse PN5–PN14 (94). Models using oxygen exposure during alveolar development may therefore better resume the characteristic picture of the so called “old BPD,” the scarring lung disease seen in infants born in late gestation in the pre-surfactant era. The process of organ maturation clearly is a modifier of the pulmonary response to oxygen exposure, as hyperoxia has been shown to reduce bone marrow, circulating and lung endothelial progenitor cells in the developing but not in the adult mouse lung (95). Additionally, different oxygen concentrations affected lung development and the host response to influenza A virus in neonatal mice (96). Hence, a better understanding of how dose and duration of the respective harmful agent interfere with a certain developmental window is important to make progress in the development of treatment strategies that could improve pulmonary health in preterm infants.

## Long-Term Consequences

Minimizing long-term pulmonary impairment, and neurologic complications associated with BPD has become the main focus of perinatal care (97, 98). Nonetheless, increasing evidence suggests that the early pathologic changes observed in BPD contribute to long-lasting consequences including premature aging of the lung.

Although only some affected infants remain oxygen dependent beyond 2 years of age, oxygen dependency for months or years is frequently described (99, 100) indicating the most severe lung disease. These infants require hospital readmission twice as often compared to infants who are not oxygen dependent. Even after having outgrown the need for oxygen supply, high readmission rates remain common in infants with BPD, with up to 70% requiring a hospital stay in the first 2 years of life (101). Here, infections with respiratory syncytial virus are the major cause for readmission among preterm infants regardless of BPD status (102). Furthermore, patients with moderate or severe BPD suffer more frequently from episodes of wheezing and need for inhalation therapies (12). Up to 80% of these infants demonstrate airway obstruction in early childhood and adolescence, with the majority being symptomatic (103–105). About 20–30% of infants with BPD suffer from those symptoms at 6 and 12 months of age (106, 107) and respiratory symptoms remain common at preschool and school age (99, 108). The most severely affected children remain symptomatic into adulthood (109). The described clinical symptoms are underlined by changes in pulmonary function as identified by clinical studies, suggesting that the presence of persistent airflow limitations and reduction in alveolar surface area as well as impaired lung growth, significantly lower peak oxygen consumption, forced expiratory volume at 1 s (FEV_1_), and gas transfer at school age after premature birth are accompanied by significantly lower peak workload, higher respiratory rates in combination with lower tidal volumes during peak exercise (110) as well as lower lung volumes and decreased gas mixing efficiency during infancy in BPD patients (111, 112).

As young adult BPD survivors present with a reduced maximal airway function and some even show a trend towards an early and steeper decline in lung function with age, the concern was raised that BPD may be a precursor of a COPD-like phenotype later in life (113) although the incidence has yet to be defined in this patient cohort (114).

With respect to the pathophysiologic concepts discussed, the effect of injury mechanism and treatment regimen has to be considered in the context of different stages in pulmonary development. Here, the inhibition of secondary alveolar formation in patients with BPD could result in premature lung structure degradation with age. Furthermore, the sustained and potentially irreversible reorganization of the ECM as observed after mechanical ventilation, oxygen exposure or steroid therapy may affect its function as a scaffold for lung development as well as its long-term repair potential, both resulting in long-term consequences. Latest studies have shown a “memory function” of the ECM as the fate of different pulmonary cell types re-populating the pulmonary scaffold was defined by its composition (115, 116). With respect to the lung vasculature, changes in angiogenic growth factor expression resemble the pattern observed in aged mice and are associated with reduced plasticity of the lung capillaries, potentially leading to sustained changes for lung development and injury response through life (117, 118). In line with this, oxygen exposure in the first week of life (FiO_2_ 1.0) has been shown to increase mortality by inducing pulmonary vascular disease in mice (119). Again, maturational differences need to be considered and studied in more detail with respect to their initial impact and their consequences for aging related processes. Whereas oxygen enhances lung vascular and airway smooth muscle contraction and reduces nitric-oxide relaxation in the neonatal rat lung, the opposite occurs in the adult animal (120). These changes are associated with altered lung function and right ventricular hypertrophy at 10 months of age, indicating significant pulmonary hypertension. At this late stage, bone morphogenetic protein signaling is altered and may contribute to the cardiovascular phenotype observed in the adult lung. Furthermore, recent studies suggest that long-term respiratory abnormalities after preterm birth may be associated with a sustained alteration of the oxidative stress response. Here, adolescent BPD patients show evidence of heightened oxidative stress in the airways (121). Likewise, the early interference with different transcription factors disrupts normal lung morphogenesis in fetal life (122), subsequently resulting in pulmonary emphysema in adult mice preluded by severe chronic bronchial inflammation (123). Underlining these findings, studies showed that suppression of the nuclear factor kappa B (NF-κB) worsened pathophysiologic changes that potentially lead to BPD development, e.g., impaired alveolarization and vascularization (122, 124, 125). Studies focusing on the differential response to injury in the developing lung will help to broaden the understanding of identified targets and processes with a critical role in adult lung diseases.

With respect to early treatment regimen, different findings indicate the need for a careful investigation of their potential to induce long-term effects. As an example, the prenatal administration of betamethasone, although widely used to enhance lung maturation thereby preventing respiratory distress and reducing BPD rates (126, 127), has been shown to be associated with an increase in lipid membrane peroxidation (89). In addition, postnatal dexamethasone treatment in the neonate, besides its negative effects on neurologic and alveolar development, led to systolic dysfunction and reduced life expectancy in elderly rats (128, 129). Furthermore, the broad use of antibiotic treatment in the mother at risk for premature delivery leads to a sustained alteration of the bacterial flora of the child (130), affecting immune function long-term as shown in neonatal in contrast to adult-germ free mice (131).

Some therapeutic agents with suggested immediate effects on the redox system, displayed their treatment potential more with respect to middle and long-term effects (132, 133). Although improving oxygenation and pulmonary vascular development in preterm sheep, intra-tracheal administration of recombinant human CuZn superoxide dismutase did not diminish the incidence of BPD, but reduced respiratory morbidity at 1 year of age and the incidence of retinopathy (ROP) (134–136). As inflammatory cells recruited to the sites of oxidative damage or attracted during infections are very likely to contribute to disease development, a combination of anti-oxidant and anti-inflammatory therapies may be most efficacious for the treatment of the structural and functional immature lung in the preterm infant. Here, studies in newborn mice showed that blocking neutrophil influx using anti-CINC antibody in diminished hyperoxia-induced DNA damage and alveolar simplification (137, 138).

As oxygen is a leading risk factor but as well remains the main treatment option in infants suffering from impaired lung growth when diagnosed with BPD, the definition of the adequate dose for oxygen therapy will remain of critical importance. Several clinical trials have attempted to identify adequate therapeutic oxygen concentrations providing maximal benefit with minimal harm. Early trials found that the incidence of ROP correlated with unrestricted use of oxygen (139) and the benefits of oxygen-saturation targeting (BOOST) trial found that a higher oxygen-saturation range prolonged oxygen dependence (140). However, the surfactant, positive pressure, and pulse oximetry randomized trial (SUPPORT) showed in 1300 infants between 24 and 28 weeks gestation treated with low oxygen saturation (85–89%) to have reduced ROP but higher mortality compared to infants with high (91–95%) oxygen-saturation target (141). These findings were confirmed in the BOOST II trial, which enrolled 2400 infants at 54 hospitals in United Kingdom, Australia, and New Zealand (142). Finally, the supplemental therapeutic oxygen for pre-threshold retinopathy of prematurity (STOP-ROP) trial found high oxygen-saturation levels did not increase the severity of ROP in those infants with pre-threshold ROP (143). Taken together, preterm infants are likely to be treated with high oxygen saturations despite the increased risk for ROP. With respect to its effect on lung function, infants with the highest quartile of oxygen exposure as a cumulative dose of inhaled gas over the first 3 days of life were two to three times more likely to experience symptomatic airway dysfunction than infants in the lowest quartile (144). In line with this quantifying cumulative oxygen exposure in newborn mice can successfully predict an altered host response of adult mice infected with influenza A virus infection (145). These findings suggest that the quantification of FiO_2_ may be a better indicator of oxygen toxicity and risk for long-term respiratory morbidity than pAO_2_ or days on oxygen therapy.

**Figure 2:**
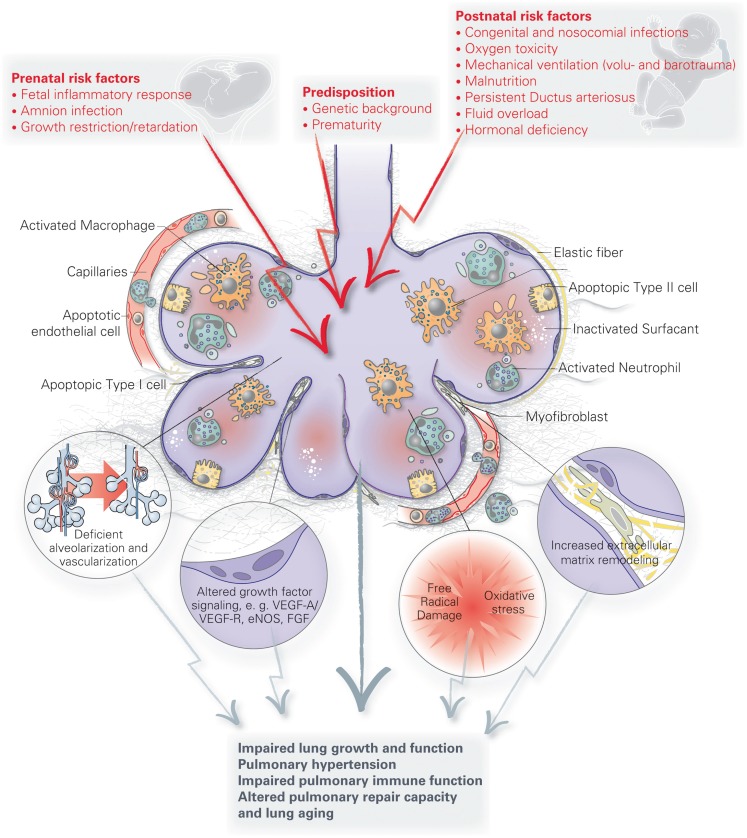
**Summary of important pathophysiologic processes and long-term consequences following early pulmonary injury**.

Figure 2 summarizes some important pathophysiologic processes and long-term consequences following early pulmonary injury dicussed here.

In order to consequently evaluate existing and further develop new treatment strategies, significant findings from disease models in rodents, allowing for the comprehensive investigation of pathophysiologic processes and long-term effects, need to be re-evaluated in animal models that very closely mimic clinical conditions in the preterm infant. Here, the premature baboon model, originally developed in the pre-surfactant era, has been shown to be very meaningful in the investigation of treatment strategies including antenatal exposure to maternal glucocorticoids, postnatal surfactant treatment, and assisted ventilation with more modest inflating pressures and concentrations of inspired oxygen (146). A similar strategy was followed in the studies performed in premature lambs (57, 81, 146–155). These studies in larger animals were integral in allowing mechanistic insight into the pathology of BPD, and permitting the evaluation of therapeutic strategies currently used in the care of premature infants, including surfactant replacement therapy (156, 157), high-frequency oscillatory ventilation (HFOV) (158), and nitric oxide (152). Further modification of these models to allow for the investigation of long-term effects as well as the evaluation of results obtained from clinical trials may provide more insight into the pros and cons of current and future treatment strategies.

To conclude, the unique response to injury observed in the immature lung including the effects on oxidative stress, ECM composition, growth factor signaling, and the sustained inflammatory response translate into a characteristic histophathologic picture. Furthermore, these changes rather contribute to a pulmonary “memory effect” than being erased over time. We now understand that early organ injury in the lung provokes pulmonary long-term consequences including alteration of physiologic aging processes as well as a characteristic response to challenges imposed on the adult lung. These considerations as well as the possible impact of an injured lung on the process of extra-pulmonary organ developmental, i.e., the brain have to be taken into account, when treatment strategies are designed and life-style issues are advocated to this patient population. Furthermore, the translation of the indicated findings into other fields of lung research and back holds great potential to inspire future ideas for the process of repair and re-programing in the diseased neonatal and adult lung.

## Conflict of Interest Statement

The authors declare that the research was conducted in the absence of any commercial or financial relationships that could be construed as a potential conflict of interest.
